# Effect of advanced nursing care on psychological disorder in patients with COVID-19: A protocol of systematic review

**DOI:** 10.1097/MD.0000000000021026

**Published:** 2020-07-02

**Authors:** Wen-juan Fan, Xiao-ling Liu

**Affiliations:** a Department of Pediatrics; b Department of Nursing Care, Yan’an People's Hospital, Yan’an, China.

**Keywords:** advanced nursing care, COVID-19, effect, psychological disorder, safety

## Abstract

**Background:**

This study will explore the effect of advanced nursing care (ANC) on psychological disorder (PD) in patients with Coronavirus Disease 2019 (COVID-19).

**Methods:**

This study will search the following electronic databases up to June 30, 2020: Cochrane Library, PUBMED, EMBASE, PsycINFO, Web of Science, OpenGrey, Cumulative Index to Nursing and Allied Health Literature, CNKI, and WANGFANG. We will not impose any language limitations. Two authors will independently identify titles/abstracts and full-text of all potential studies, and will collect data from eligible studies. Additionally, study quality will be assessed by Cochrane risk of bias. If necessary, we will conduct meta-analysis if sufficient trials are included.

**Results:**

This study will explore the effect of ANC on PD in patients with COVID-19 through outcome indicators.

**Conclusion:**

The findings of this study may supply summarized evidence of ANC for the management of PD in COVID-19.

**PROSPERO registration number:**

PROSPERO CRD42020187610.

## 1 Introduction

Coronavirus Disease 2019 (COVID-19) is a rapid, global spread virus,^[1–4]^ which attacks people in almost all countries worldwide.^[5–8]^ So far, only its transmission in China has been controlled.^[9,10]^ The rate of its infections outside China is still rapidly increasing, especially in the United States, which accounts for almost one-third of all patients with COVID-19 globally.^[11,12]^ Till May 25, 2020, about 5,586,715 cases of COVID-19 and 347,852 deaths have been reported.^[12]^ A variety of studies reported that patients who received or even cured with this condition often experience psychological disorder (PD), including depression, and anxiety.^[13–24]^ So the effective prevention and treatment of PD in patients with COVID-19 are very essential task. Studies suggested that advanced nursing care (ANC) may benefit PD in patients with COVID-19.^[25–28]^ However, no systematic review has investigated this issue. Therefore, this study will aim to assess the effect of ANC on managing PD in patients with COVID-19.

## 2 Methods

### 2.1 Study registration

This study was registered through PROSPERO (CRD42020187610). It is reported in accordance with the guidelines of Preferred Reporting Items for Systematic Reviews and Meta-Analysis Protocol statement.

### 2.2 Eligibility criteria

#### 2.2.1 Study types

The present study will include randomized controlled trials (RCTs) on investigating the effect of ANC on PD in patients with COVID-19, irrespective language, and publication status.

#### 2.2.2 Intervention types

In the experimental group, all patients received ANC on PD in patients with COVID-19.

In the control group, no restrictions related to the comparators are applied. However, we will not consider any controls involved in ANC.

#### 2.2.3 Participant types

This study will include patients with COVID-19, who were diagnosed as PD, regardless sex, race, age, and severity of PD.

#### 2.2.4 Outcome measurements

Primary outcomes are depression and anxiety, as measured by any related scales reported in the primary trials.

Secondary outcomes are pressure, panic disorder, quality of life, and expected and unexpected adverse events.

### 2.3 Search strategy

We will search following electronic databases (Cochrane Library, PUBMED, EMBASE, PsycINFO, Web of Science, OpenGrey, Cumulative Index to Nursing and Allied Health Literature, CNKI and WANGFANG) up to June 30, 2020 without language and publication status restrictions. In addition, gray literatures will also be searched, including conference proceedings, reference lists of included studies, and websites of clinical trial registry. All potential RCTs of ANC on PD in patients with COVID-19 will be considered. The specifics of Cochrane Library are presented in Table 1. We will modify similar search strategies to the other electronic databases.

**Table 1:**
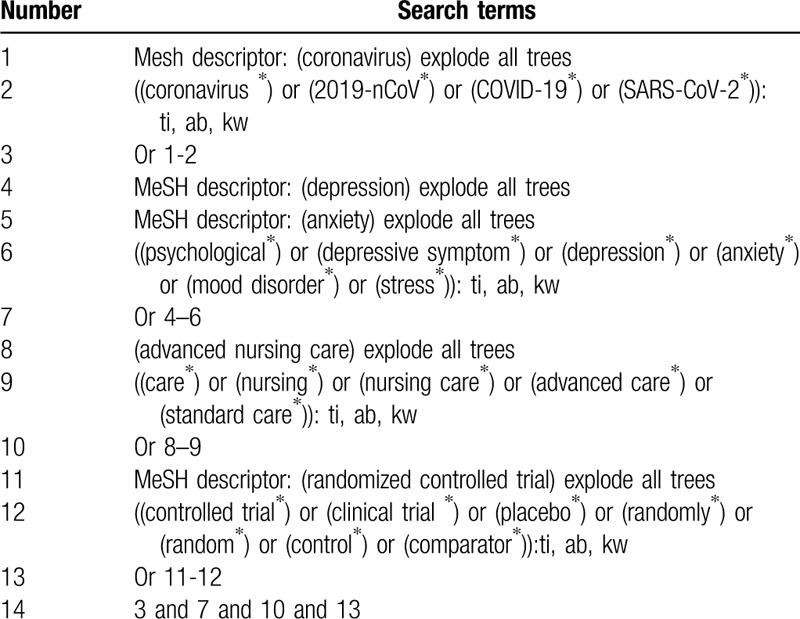
Search strategy for Cochrane Library.

### 2.4 Study selection

Two authors will independently scan the retrieved records based on titles/abstracts; and unrelated studies will be excluded. Then, we will read full articles of remaining studies in accordance with the full inclusion criteria. Each excluded study will be recorded with specific reason. The whole process of study selection will be shown in a flow chart. If any divergences occur, we will invite a third author to help solve them through discussion.

### 2.5 Data collection and management

Two authors will independently collect data from all eligible studies based on the data extraction form. It consists of following information: title, publication time, country, study design and setting, participant characteristics, eligible criteria, treatments and controls, outcomes, risk of bias, and other essential information. We will solve any different opinions by inviting another experienced author through discussion.

### 2.6 Missing data dealing with

Whenever the missing or unclear data are identified, we will contact original trial authors to request them. Otherwise, we will analyze the available data if we cannot achieve them.

### 2.7 Risk of bias assessment

We will utilize Cochrane risk of bias tool to evaluate risk of bias for each included trial. Two authors will independently appraise it through 7 domains. We will make a judgment for each item using 1 of 3 categories (high, unclear, and low risk of bias). We will solve disagreements by consulting a third author.

### 2.8 Data synthesis

We will use RevMan 5.3 software to perform statistical analysis. All continuous data will be estimated as standardized mean difference and 95% confidence intervals (CIs), and all dichotomous data will be expressed as risk ratio and 95% CIs. All statistical heterogeneity is appraised by *I*^*2*^ test. *I*^*2*^ ≤50% indicates minor heterogeneity, and a fixed-effect model will be applied. *I*^*2*^ ≤50% suggests obvious heterogeneity, and a random-effect model will be placed. If sufficient data are collected with minor heterogeneity, we will plan to conduct meta-analysis. If remarkable heterogeneity is detected, we will carry out subgroup analysis to test its heterogeneity sources. If necessary, we will report study results by narrative description.

### 2.9 Subgroup analysis

We will perform subgroup analysis according to the different details of interventions, study quality and outcome indicators.

### 2.10 Sensitivity analysis

We will undertake sensitivity analysis to examine the robustness and stability of synthesized results by eliminating low-quality studies.

### 2.11 Reporting bias

We will detect reporting bias using Funnel plot and Egger regression test if >10 eligible studies are included.

### 2.12 Ethics and dissemination

No ethics approval is required, since no individual patient data will be collected in this study. We will publish this study at a peer-reviewed journal.

## 3 Discussion

COVID-19 is a globally spread disease, which attacks almost all countries.^[1–4]^ Studies reported that patients who experience or cured with COVID-19 often have PD. Thus, it is very important to manage such condition. Several studies reported that ANC can help patients with this disorder. However, no systematic review assessed its effects. Thus, this study is the first one to systematically and comprehensively appraise the effects of ANC on PD in patients with COVID-19. The results of this study may provide evidence to determine whether ANC is effective for the treatment of PD following COVID-19.

## Author contributions

**Conceptualization:** Wen-juan Fan, Xiao-ling Liu.

**Data curation:** Wen-juan Fan, Xiao-ling Liu.

**Formal analysis:** Wen-juan Fan, Xiao-ling Liu.

**Investigation:** Xiao-ling Liu.

**Methodology:** Wen-juan Fan.

**Project administration:** Xiao-ling Liu.

**Resources:** Wen-juan Fan.

**Software:** Wen-juan Fan.

**Supervision:** Xiao-ling Liu.

**Validation:** Wen-juan Fan, Xiao-ling Liu.

**Visualization:** Wen-juan Fan, Xiao-ling Liu.

**Writing – original draft:** Wen-juan Fan, Xiao-ling Liu.

**Writing – review & editing:** Wen-juan Fan, Xiao-ling Liu.
